# Global analysis of the *Gossypium hirsutum* L. Transcriptome during leaf senescence by RNA-Seq

**DOI:** 10.1186/s12870-015-0433-5

**Published:** 2015-02-12

**Authors:** Min Lin, Chaoyou Pang, Shuli Fan, Meizhen Song, Hengling Wei, Shuxun Yu

**Affiliations:** State Key Laboratory of Cotton Biology, Institute of Cotton Research, Chinese Academy of Agricultural Sciences, Anyang, Henan 455112 China

**Keywords:** Cotton, Transcriptome, Leaf senescence, Transcription factors, Hormone

## Abstract

**Background:**

Leaf senescence is an important developmental programmed degeneration process that dramatically affects crop quality and yield. The regulation of senescence is highly complex. Although senescence regulatory genes have been well characterized in model species such as *Arabidopsis* and rice, there is little information on the control of this process in cotton. Here, the senescence process in cotton (*Gossypium hirsutum* L.) leaves was investigated over a time course including young leaf, mature leaf and leaf samples from different senescence stages using RNA-Seq.

**Results:**

Of 24,846 genes detected by mapping the tags to *Gossypium* genomes, 3,624 genes were identified as differentially expressed during leaf senescence. There was some overlap between the genes identified here and senescence-associated genes previously identified in other species. Most of the genes related to photosynthesis, chlorophyll metabolism and carbon fixation were downregulated; whereas those for plant hormone signal transduction were upregulated. Quantitative real-time PCR was used to evaluate the results of RNA-Seq for gene expression profiles. Furthermore, 519 differentially expressed transcription factors were identified, notably WRKY, bHLH and C3H. In addition, 960 genes involved in the metabolism and regulation of eight hormones were identified, of which many genes involved in the abscisic acid, brassinosteroid, jasmonic acid, salicylic acid and ethylene pathways were upregulated, indicating that these hormone-related genes might play crucial roles in cotton leaf development and senescence. However, most auxin, cytokinin and gibberellin pathway-related genes were downregulated, suggesting that these three hormones may act as negative regulators of senescence.

**Conclusions:**

This is the first high-resolution, multiple time-course, genome-wide comprehensive analysis of gene expression in cotton. These data are the most comprehensive dataset currently available for cotton leaf senescence, and will serve as a useful resource for unraveling the functions of many specific genes involved in cotton leaf development and senescence.

**Electronic supplementary material:**

The online version of this article (doi:10.1186/s12870-015-0433-5) contains supplementary material, which is available to authorized users.

## Background

Leaf senescence is the ultimate phase of plant leaf development, and involves coordinated action at the cell, tissue, organ and organism levels. Senescence is controlled by a highly regulated genetic program and leads to death or the end of the life span [1]. Leaf senescence is not a passive and unregulated degeneration process; during senescence leaf cells undergo dramatically coordinated changes in cell structure, metabolism and gene expression [2]. The earliest and most significant cellular degeneration process begins with the chloroplast, which contains most of the proteins in a leaf cell. The mitochondria and nucleus remain intact until the final stages of leaf senescence. Metabolically, carbon assimilation, such as photosynthesis, is replaced by catabolism of chlorophyll and macromolecules, such as proteins, membrane lipids and nucleic acids [3]. Although leaf senescence is a deleterious process, it is crucial for the fitness of plants; efficient senescence is essential to maximize viability during the plant’s life cycle. Senescence can also occur prematurely when plants are stressed; it is a protective mechanism, leading to decreased yield and quality in crop plants by limiting the growth phase, which has become an increasing concern because of global climate change in recent years [4].

Recently, many advances have been made in understanding leaf senescence at the molecular level by the identification and characterization of a large number of senescence-associated genes (SAGs) and senescence-related mutants in various plant species, including plants such as *Arabidopsis thaliana*, *Oryza sativa*, and *Medicago truncatula* [5-10]. Among these SAGs, numerous transcription factors (TFs) such as NAC, WRKY, MYB [11], kinases and receptor-like kinases [12], signal transduction-related proteins, and regulators of metabolism are involved in regulating leaf senescence, indicating that senescence is an integrated response to many signals that are governed by highly complex transcriptional regulatory networks.

Cotton (*Gossypium hirsutum* L.) is one of most important economic crops, and is widely cultivated for the value of its fiber. In cotton, leaf senescence may occur too early or too late under the influence of certain internal factors or uneven environmental stresses [13]. Late senescence would affect remobilization of nutrients from the leaf to newly formed sink organs, such as developing boll. Whereas, premature senescence has occurred with increasing frequency since the introduction of modern, high-yielding cotton cultivars, such transgenic Bacillus thuringiensis (Bt) cotton, where premature senescence frequently developed during the period of rapid boll filling. Premature senescence results in reduced lint yield and poor fiber properties [14,15]. Previous studies on leaf senescence in cotton were limited mainly to physiological and cytological studies. The initiation and progression of leaf senescence in cotton can be affected by internal factors, such as phytohormones, mainly cytokinin (CK) and abscisic acid (ABA) [16], or various environmental factors such as drought [17], nutrient deficiency [15,18], salinity [19], UV-B radiation and elevated CO_2_ [20,21]. However, the mechanisms and the global transcriptional regulation of leaf senescence in cotton remain poorly understood.

To date, many molecular approaches have been used to understand the biochemical pathways and regulatory mechanisms associated with leaf senescence. Many novel SAGs were identified with distinct expression profiles during *Arabidopsis* developmental leaf senescence and induced senescence; comparison of changes in gene expression patterns in different senescent systems indicated differences in the genetic programs, such as nitrogen mobilization [12,22]. A DNA microarray comprising 13,490 expressed sequence tags was used to investigate gene expression during leaf senescence in *Populus*; a major shift in gene expression from photosynthetic competence to energy generation was observed [23]. Recently, analysis of DNA microarrays analysis showed a high-resolution time-course profile of gene expression during *Arabidopsis* leaf senescence [24]. The next-generation high-throughput sequencing technology, referred to as RNA-Seq, has emerged as a revolutionary tool to better understand complex eukaryote transcriptome [25]. In this approach, there is no strict requirement for a reference genome sequence [26]; therefore, it is suitable for the study of non-model species whose entire genomic sequences are not available. RNA-Seq has been used to investigate gene expression in many plants [27-29]. In the present study, RNA-Seq was performed on samples from different stages of leaf development, and the global changes in the developing leaf transcriptome were analyzed. Based on extensive data analyses, many genes and pathways were identified and characterized, which involved in leaf development and senescence in cotton. Analysis of TFs and hormone-related genes revealed an integrative image of gene regulatory networks. This work will serve as the foundation for further study of the molecular regulation mechanisms of leaf senescence in *G. hirsutum* and represents an invaluable resource for further identification of genes involved in leaf senescence.

## Results

### Biochemical changes during senescence and selection of the analysis period

**Figure 1:**
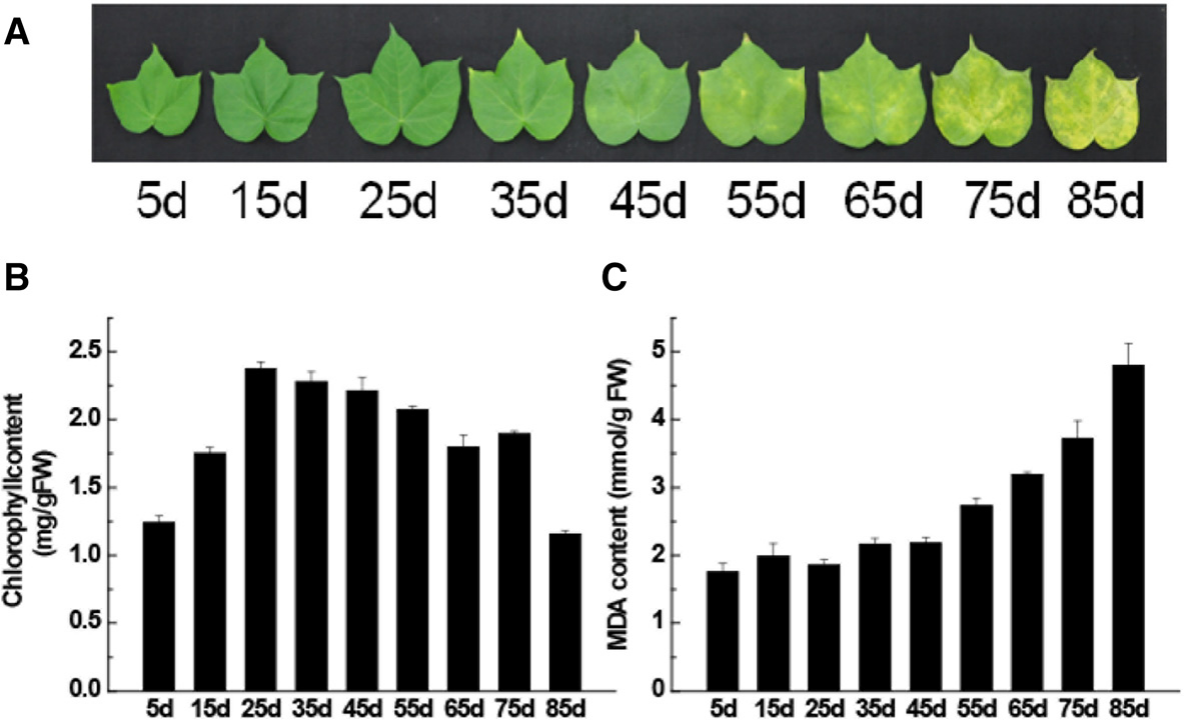
**Plant phenotypes, and chlorophyll and MDA measurements. A**. Appearance of *G. hirsutum* at eight different stages of development. **B**. Chlorophyll levels were measured in leaf samples at each stage of development. FW, fresh weight. Error bars represent the SD from three independent experiments. **C**. MDA contents were measured in leaf samples at each stage of development. FW, fresh weight. Error bars represent the SD from three independent experiments.

In this study, newly emerging cotton fourth leaves of the same size were tagged; the day when the newly emerging leaf expanded was considered as the first day. As shown in Figure 1A, leaves started to show yellowing at the tip at around 35 d and leaves were harvested at defined time points until 85 d, when they were visibly senescent (about 85% of the leaf area was yellow). Malondialdehyde (MDA) and chlorophyll levels are often used as markers for the progression of senescence because the levels of both change significantly during senescence. Levels of total chlorophyll and MDA were measured (Figure 1B,C). Chlorophyll levels increased from 5 d until 25 d, reaching a maximum at 25 d, and then declined. The MDA level did not change significantly until 55 d and then continued to increase. Based on the phenotype and physiological tests (Figure 1), leaf samples at 15, 25, 35, 45, 55 and 65 d were selected for digital gene expression (DGE) sequencing analysis.

### RNA-Seq analysis over multiple time points and the identification of differentially expressed genes (DEGs)

Six DGE libraries were sequenced, representing time points at 15, 25, 35, 45, 55 and 65 d, which generated approximately 4.8 million raw tags in each library (SRA submission number: SRR656612). After filtering out the low quality tags, the total number of clean tags per library ranged from 4.3 to 4.7 million. To reveal the molecular events underlying the DGE profiles, the tag sequences of these six DGE libraries were mapped to non-redundant reference sequence (the reference sequence is composed of 47% of *G. raimondii* genome and 53% of *G. arboretum* genome). Approximately 30% of all distinct tags could be unambiguously mapped to a gene in the reference database and 60% of the distinct clean tags were mapped to the *Gossypium* genome database. 24,846 genes were detected as expressed during the cotton leaf development; among these, 3,624 DEGs were obtained (Additional file 1).

### Confirmation of tag-mapped genes by qRT-PCR

**Figure 2:**
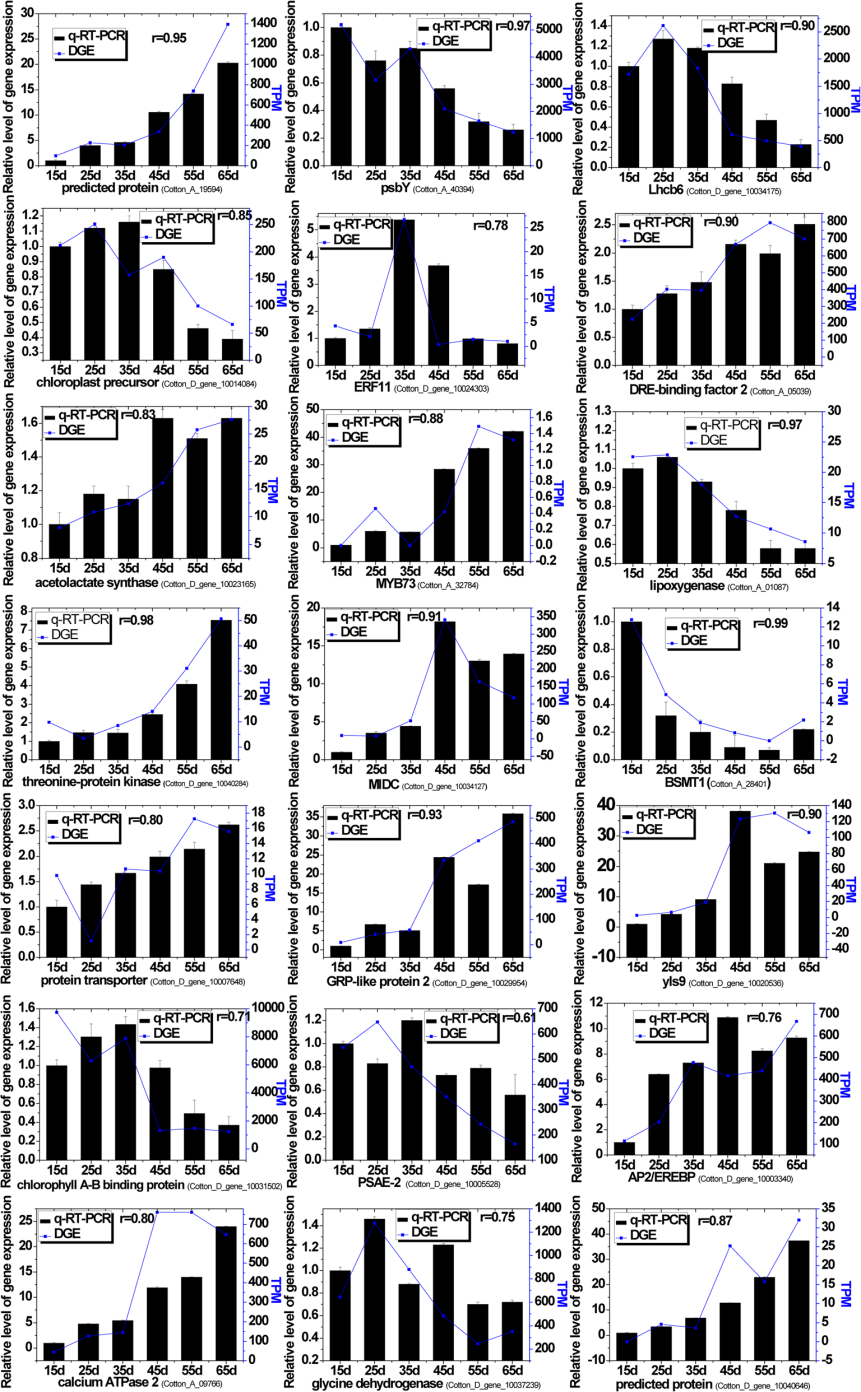
**qRT-PCR validation of selected transcripts in cotton leaves.** The columns represent the expression level of the transcript (left y-axis); the lines represent the relative intensity of real-time qRT-PCR from independent biological replicates (right y-axis).

To validate the results of the gene expression analysis obtained using DGE data, qRT-PCR was performed on selected genes with different expression levels and functional assignments. Transcriptional regulation revealed by RNA-Seq was confirmed in a biologically independent experiment using qRT-PCR. Of the 25 candidates, four genes showed either no specific amplification or unexpected size amplification. The Pearson correlation coefficient (calculated by SAS software) was used to assess the correlation between different platforms. Overall, the outcome of qRT-PCR was in very good agreement (the mean correlation coefficient of 0.86) with the DGE results (Figure 2). Thus, our data demonstrated that the DGE technique for counting transcripts gives an accurate reflection of transcript abundance, and can be used for gene expression analysis in an organism lacking genome information.

### Comparison of SAGs between cotton and other plants

To analyze SAG orthologs between cotton and other plants, identified DEGs were compared with those in other species in the leaf senescence database (LSD, June, 2013; http://www.eplantsenescence.org/) using BLASTX searches (e-value ≤10^−10^). These genes were grouped into several categories according to the function and classification of the most closely related homolog. 2, 414 out of the 3,624 DEGs have homologous genes in other plants in the LSD. Most of the genes were homologous to genes in *Arabidopsis*, closely followed by Banana. Interestingly, these orthologs were mainly involved in transcriptional regulation, protein degradation/modification, signal transduction, redox regulation, lipid/carbohydrate metabolism, nutrient recycling, hormone response pathway and transport (Additional file 2).

### Clustering of DEGs by expression pattern illustrated metabolic changes occurring during leaf senescence

**Figure 3:**
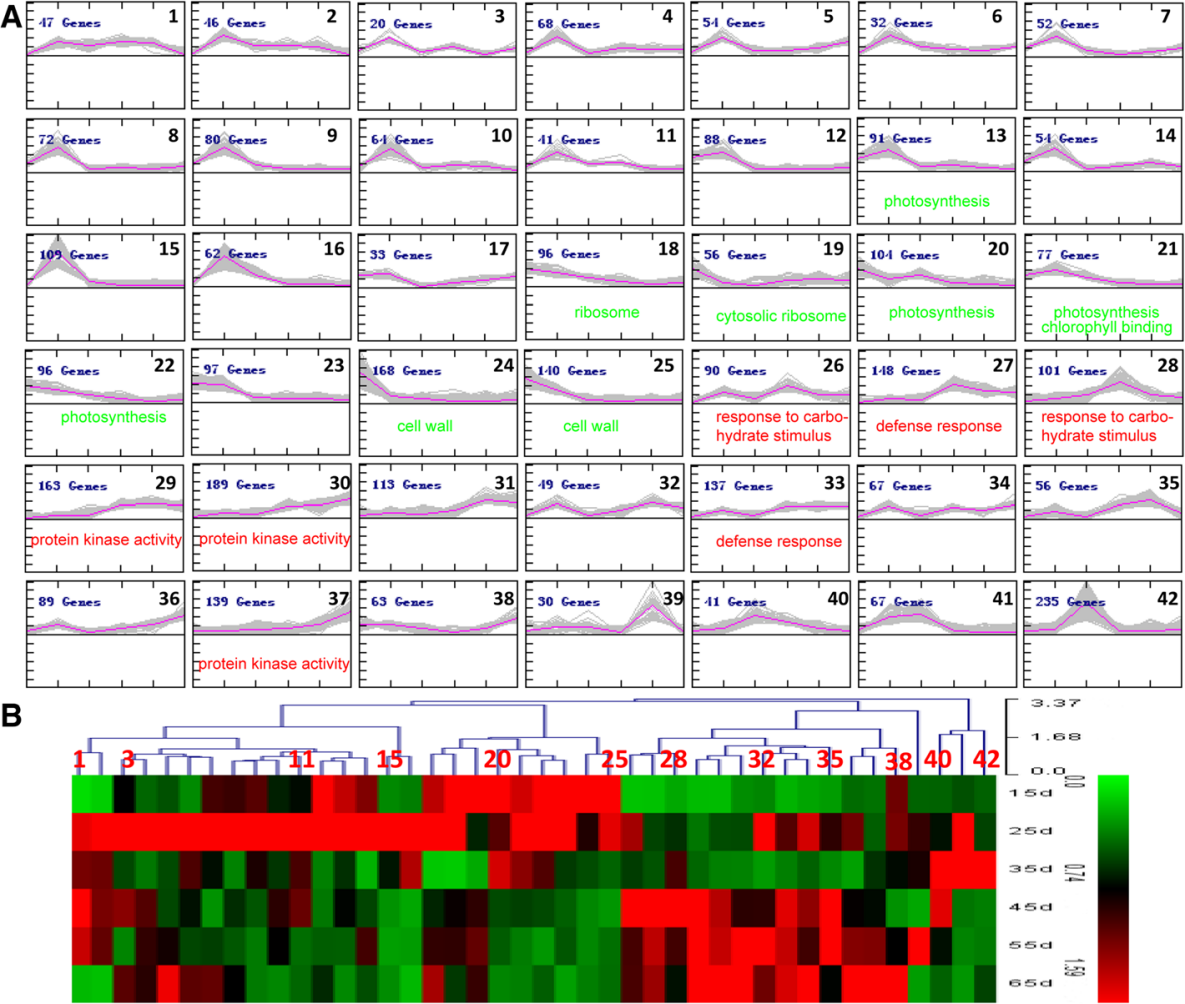
**MeV cluster analysis of differentially expressed genes. A**. Cluster analysis by the K-means method from the gene expression profiles. Selected enriched gene ontology (GO) terms (data shown in Additional file 3) are indicated; green indicates downregulated and red indicates upregulated genes. **B**. Heat map illustrating the expression profiles of the genes in each cluster.

Genes with similar expression patterns are often functionally correlated. To screen novel candidate genes whose functions correlate with leaf senescence, the 3,624 DEGs were clustered by MultiExperiment Viewer (MeV, v4.7.4), based on the K-means method and hierarchical clustering, respectively. Forty-two clusters were obtained for the data for the six time points (Figure 3A), the cluster number and corresponding expression values for each DEG in the six time point clusters are shown in Additional file 1. A heat map (Figure 3B) was generated to illustrate the changes in gene expression at each time point. There are several time points at which an obvious step change in the transcriptome occurs. Overall, the major switch in gene expression, both in upregulated and downregulated genes, occurs between 25 d and 35 d. Similarly, the genes identified as differentially expressed can be divided into two major groups based on their expression patterns (Figure 3). Type I includes 1,847 genes from clusters 1 to 25 that were downregulated during this period, and Type II includes 1,434 genes from clusters 26 to 39 that were upregulated. Some of the clusters showed a more complex pattern; for example, genes in clusters 40, 41, and 42 showed an initial increase followed by a decrease in expression (Figure 3).

**Figure 4:**
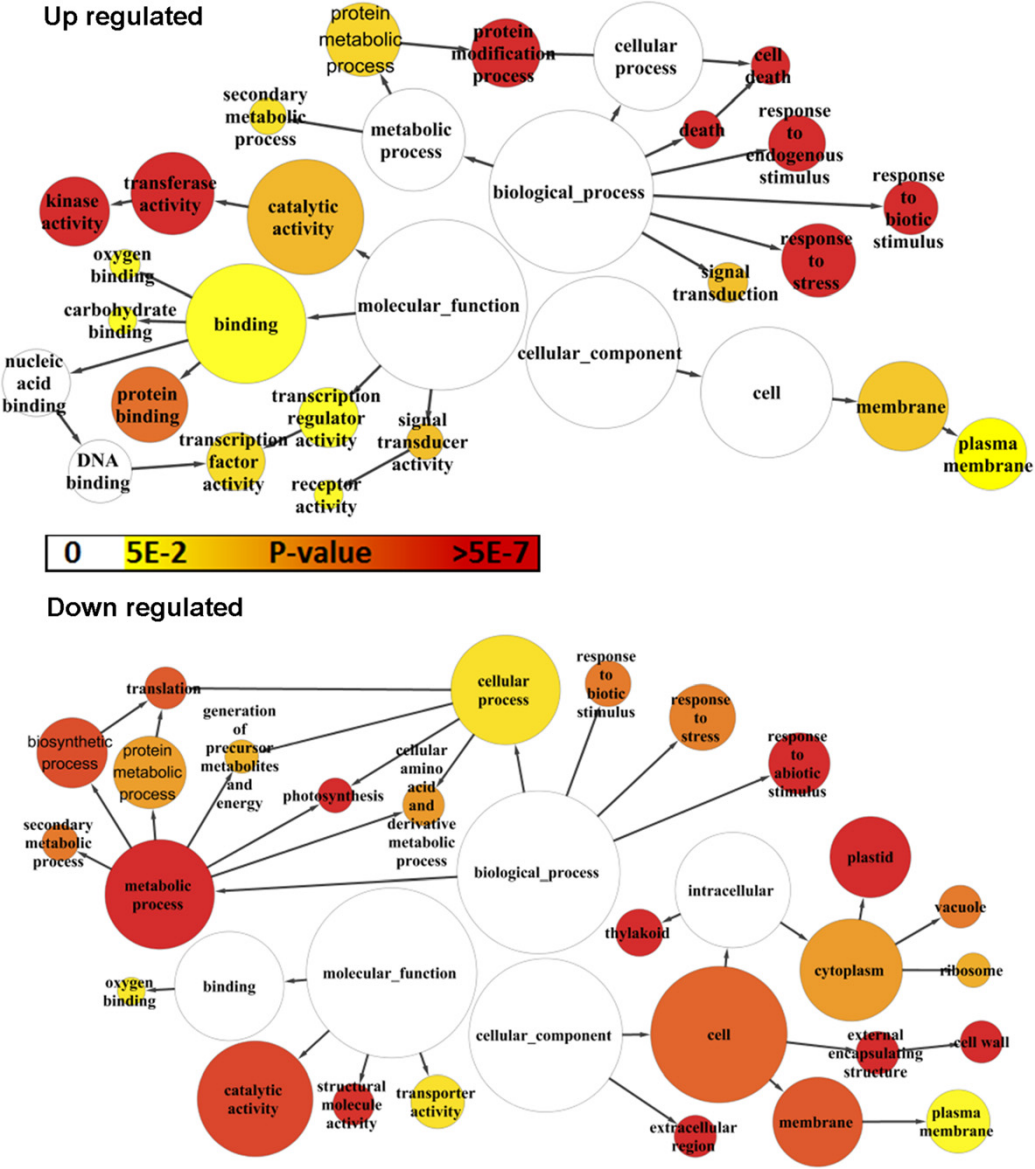
**GO terms enriched for genes upregulated and downregulated during leaf Senescence.** The network graphs show BiNGO visualization of the overrepresented GO terms for the combined clusters of genes either upregulated or downregulated during senescence. Colored nodes represent GO terms that are significantly overrepresented; the colors are shaded according to the significance level as shown in the color bar.

To permit the global analysis of gene expression profiles and reveal the major functional categories represented in the DEGs during cotton leaf development, the two groups of genes showing either decreasing (clusters 1–25) or increasing (clusters 26–39) expression during leaf development were analyzed by GO enrichment tool BiNGO [30]. The BiNGO-derived graph (Figure 4) indicated the most highly significant enrichment of specific functions. Downregulated genes were significantly enriched for genes linked to cellular components such as thylakoid, plastid, vacuole, membrane, cell wall and extracellular region, and there was significant enrichment for photosynthesis, biosynthetic process, generation of precursor metabolites and energy in the biological process term. Within the molecular function category, catalytic activity and structural molecule activity were overrepresented. All these functions are essential for leaf growth, but are downregulated during senescence. The loss of integrity of cellular structures, such the membrane, thylakoid, plastid and cell wall leads to disruption of cellular homeostasis, ending the life of a cell in senescing leaves. The upregulated genes showed a very different picture, the most significantly overrepresented terms focused on the defense and protective steps that the plant takes to respond to the stress generated by a series of degradative processes and nutrition recycling. Within the biological process, response to endogenous stimulus, response to biotic stimulus, cell death, response to stress, protein modification process and signal transduction were overrepresented. For the molecular function, kinase activity, transferase activity, transcription factor activity and protein binding are overrepresented, whereas few cellular component terms are overrepresented. Meanwhile, to investigate the dynamic changes in gene expression and associated metabolic process, enriched GO terms in individual clusters were also analyzed (Figure 3A and Additional file 3). Unsurprisingly, chlorophyll biosynthesis genes and photosynthesis-related genes were significantly enriched; particularly photosynthesis-related genes, which were downregulated in many clusters. Clusters 18 and 19 are enriched genes for ribosome and cytosolic ribosome, cluster 21 contained genes involved in chlorophyll binding. Meanwhile, cluster 24 and 25 also contained many genes associated with cell wall. For the upregulated clusters, enrichment was seen for many genes involved in response to carbohydrate stimulus, defense response and protein kinase activity.

### Several biochemical pathways are significantly enriched during leaf senescence

All DEGs were analyzed using the KEGG Orthology Based Annotation System (KOBAS) to identify the metabolic pathways in which they function. A total of 1, 769 downregulated genes mapped to 100 KEGG pathways and 1, 357 upregulated genes mapped to 88 KEGG pathways (Additional file 4). Four of these pathways were significantly downregulated, whereas five of these pathways were significantly upregulated (corrected p-value ≤ 0.05) during leaf senescence (Table 1). Many typical senescence symptoms such as photosynthesis-antenna proteins, photosynthesis, porphyrin and chlorophyll metabolism, and carbon fixation were significantly overrepresented in the downregulated pathways. Certain protective activities pathways were significantly enriched in the upregulated genes, such as plant-pathogen interaction, fatty acid elongation, glutathione metabolism, taurine and hypotaurine metabolism. Meanwhile, plant hormone signal transduction pathway was obvious activated in the upregulated genes. These results suggested that these pathways might play an important role in leaf senescence.

**Table 1:** **Significantly enriched pathways in cotton identified by KOBAS during leaf senescence (Corrected P-value ≤ 0.05)**

**KEGG id**	**KEGG pathway name (down-regulated)**	**Gene number**	**Background number**	**P-value**	**Corrected p-value**
ko00196	Photosynthesis - antenna proteins	22	31	5.70E-11	7.88E-09
ko00195	Photosynthesis	24	86	9.92E-06	4.02E-04
ko00860	Porphyrin and chlorophyll metabolism	16	65	9.33E-04	1.88E-02
ko00710	Carbon fixation in photosynthetic organisms	22	115	2.14E-03	3.88E-02
**KEGG id**	**KEGG pathway name (up-regulated)**	**Gene number**	**Background number**	**P-value**	**Corrected P-value**
ko04626	Plant-pathogen interaction	42	231	4.55E-13	1.86E-11
ko04075	Plant hormone signal transduction	34	338	2.78E-05	4.74E-04
ko00062	Fatty acid elongation	8	38	6.84E-04	9.57E-03
ko00480	Glutathione metabolism	13	99	1.03E-03	1.37E-02
ko00430	Taurine and hypotaurine metabolism	4	11	3.16E-03	3.62E-02

### TF families are active at different times during senescence

**Figure 5:**
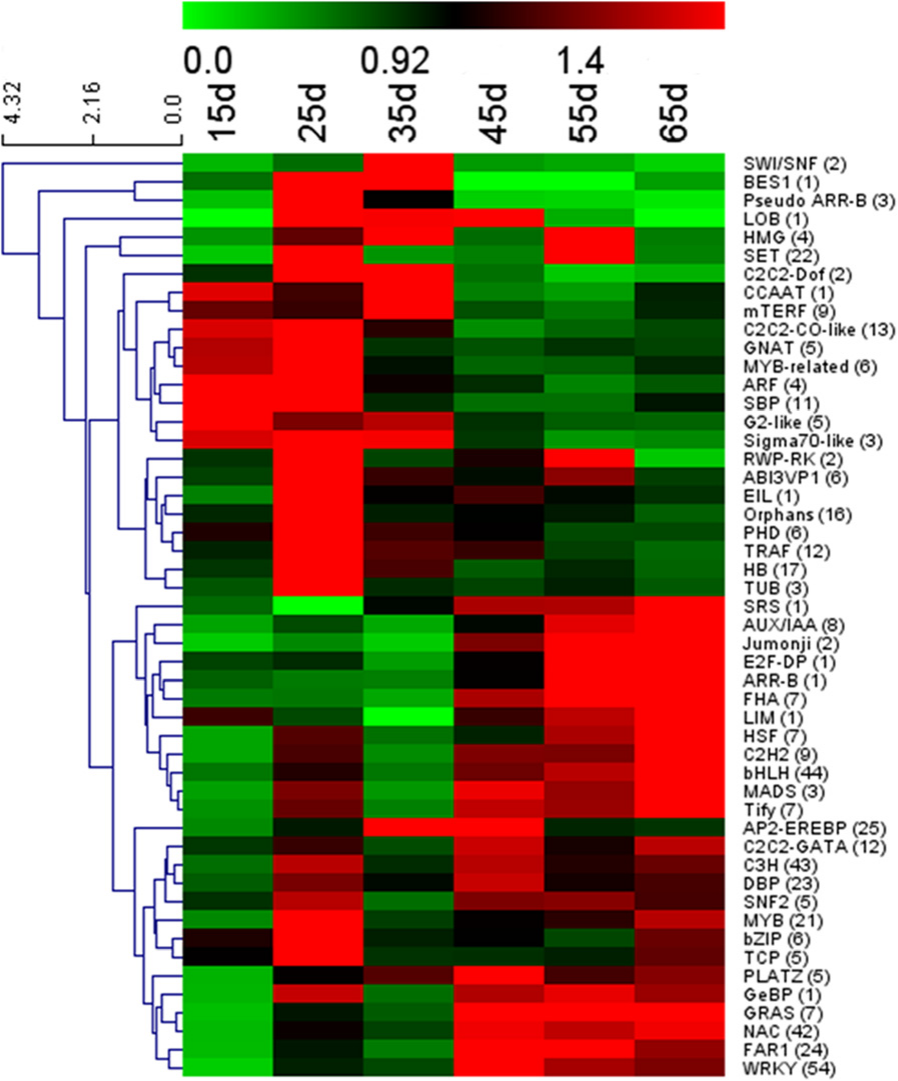
**Hierarchical cluster analyses for cotton leaf putative TFs differentially expressed during leaf senescence.** Various transcription factor families showing differential expression are shown on the right side. The x-axis refers to each stage of leaf development. The y-axis represents the expression levels of the genes. Numbers in parentheses represent the number of transcription factor family members.

TFs are key regulatory proteins that are essential for regulation of gene expression. TFs are known to play significant roles in leaf senescence. Among the 3,624 DEGs, 519 TF transcripts from 50 TF families were identified (Additional file 5). The WRKY (54), bHLH (44), C3H (43), NAC (42), AP2-EREBP (25), FAR1 (24), DBP (23), SET (22), MYB (21) and HB (17) families were the top ten largest families active during leaf development, some of which are critical components of plant adaptive response to biotic, abiotic stresses and senescence. A heat map depicting the overall trend of the differential expression profiles of the TF genes during leaf senescence was constructed using MeV. As shown in Figure 5, many TF families were significantly upregulated. For example, the large WRKY and C3H family were significantly upregulated, with many members of these families being upregulated early, from around 25 d. Another significantly overrepresented upregulated family was the large NAC family, with over 40 of the members of this family showing altered expression at various times during senescence. Many members of the NAC family have been implicated functionally in senescence and in a variety of stress-related programs [11,23,31-34]. In addition, bHLH, GRAS, MYB, DBP and AP2-EREBP TFs are differentially regulated during senescence and were also upregulated during the early stages in our data [11,22-24,32].

### Differential expression of hormone regulation-related genes during leaf Senescence

To identify the contribution of hormone-mediated transcriptional regulation to changes in patterns of gene expression during leaf senescence, we mapped the DGE transcripts to eight hormone-related pathways in the Arabidopsis Hormone Database, including ABA, auxin, ethylene (ET), brassinosteroid (BR), jasmonic acid (JA), CK, GA and salicylic acid (SA). 960 genes associated with various aspects of hormone homeostasis, such as biosynthesis, response, signaling, receptors and metabolism exhibited significant differential expression during senescence. (Table 2 and Additional file 6).

**Table 2:** **Hormone-related genes differentially expressed in leaves during leaf senescence**

**Hormones**	**Total number of genes**	**Upregulated**	**Downregulated**	**Complex**
ABA	284	154	109	21
Auxin	138	58	67	13
CK	41	14	23	4
BR	125	68	53	4
ET	78	36	34	8
GA	62	18	43	1
JA	78	43	31	4
SA	154	111	41	2

**Figure 6:**
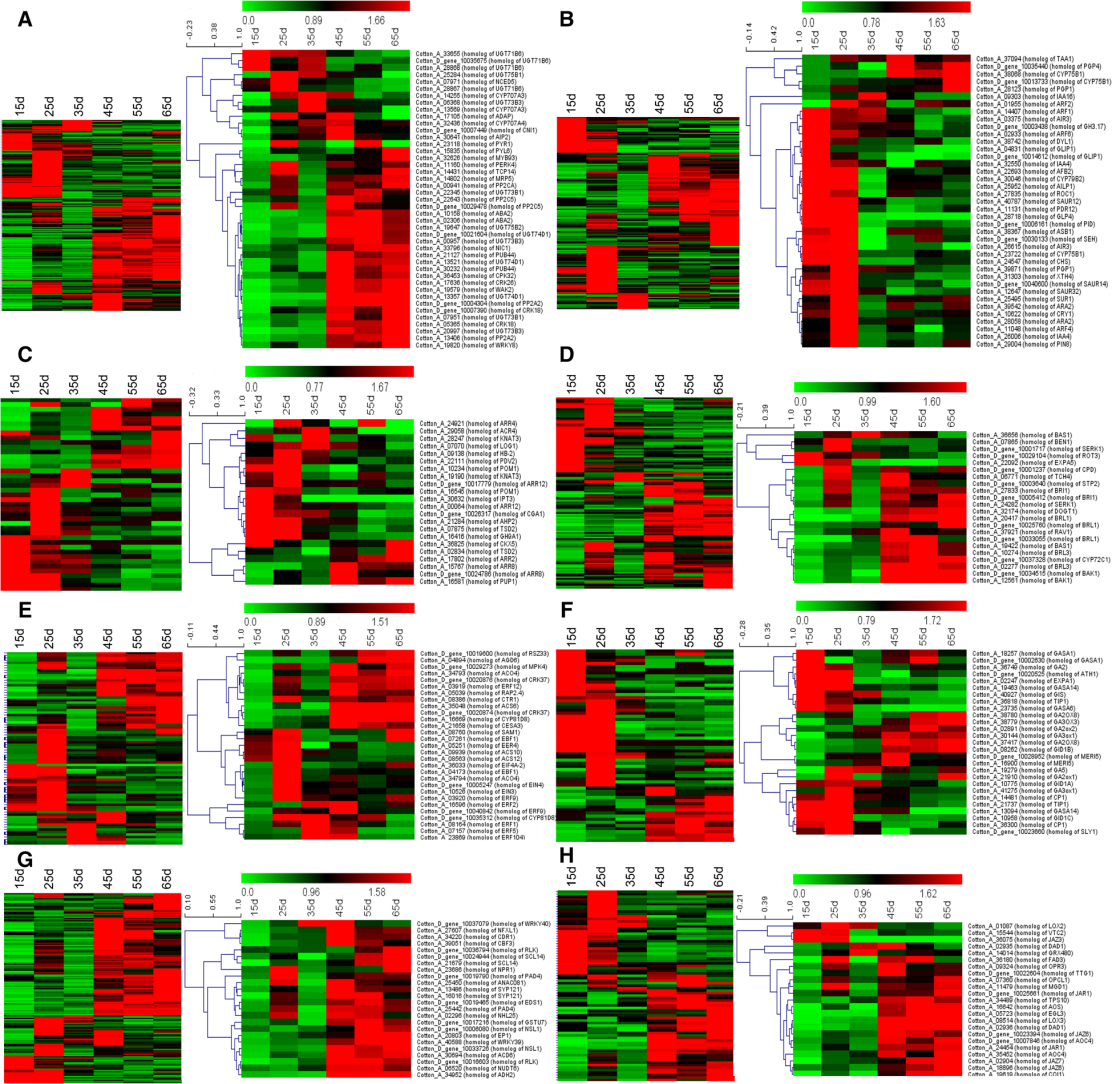
**Hierarchical cluster analysis of differentially expressed transcripts involved in each hormone pathway during senescence. A**. abscisic acid, ABA; **B**. auxin; **C**. cytokinin, CK; **D**. brassinosteroid, BR; **E**. ethylene, ET; **F**. Gibberellic acid, GA; **G**. salicylic acid, SA; **H**. jasmonic acid, JA. The left heat map illustrates the expression profiles for transcripts in the hormone pathway; the right heat map illustrates the expression profiles of transcripts mentioned in the context of hormone. The x-axis refers to each stage of leaf development. The y-axis represents the expression levels of the genes.

The largest group that showed significant differential expression was related to ABA (Figure 6A), which is involved extensively in response to environmental stresses and leaf senescence [35,36]. Several genes that are involved in ABA synthesis, response, metabolism, receptors or signaling showed differential expression. Two UDP-glucosyltransferase (*UGT*) 73B1 genes, three *UGT73B3* genes, three *UGT74D1* genes, one *UGT75B1* and one *UGT75B2* gene were significantly upregulated during senescence. Meanwhile, two *CYP707A3* homologs and one *CYP707A4* homolog were upregulated at all leaf senescence stages. However, four homologs of ABA-inactivating *UGT71B6* were downregulated during senescence. Investigation of the expressions of ABA signaling related genes showed that many genes were upregulated. For example, among protein phosphatase genes, *Arabidopsis thaliana* ABA deficient 2 (*ABA2*), which is implicated in early events of ABA signalling [37], had four homologs that were upregulated. All three ABA receptor pyrabactin resistance (*PYR*)/PYR1-like (*PYL*) homologs were upregulated. Of the fourteen homologs of protein phosphatase 2C (*PP2C*), which negatively regulates the ABA response, most homologs were upregulated during senescence. The ARIA-interacting double AP2 domain protein (*ADAP*) gene is involved in the ABA response, where it interacts with *ARIA*, which was also identified in this study. One *ADAP* homolog was upregulated. Plant U-box 44 (*PUB44*) genes (also called senescence-associated E3 ubiquitin ligase 1 homologs), which have important roles in the regulation of ABA signaling process, were also significantly upregulated.

Auxin plays important roles in the regulation of root formation, apical dominance, fruit development and senescence [38-40]. For auxin biosynthesis related genes (Figure 6B), one cytochrome P450 75B1 (*CYP75B1*) homolog and one *CYP79B2* homolog were downregulated. P-glycoprotein 1 (*PGP1*) is associated with auxin transport and is probably involved in the correct polar distribution of auxin during plant development. *PGP1* homologs were upregulated during the early senescence stage. The homolog of *PGP4*, which presumably functions as an auxin importer in root caps [41], appeared to be weakly upregulated during later stages of senescence. In addition, the homologs of putative auxin efflux carrier component 8 (*PIN8*) from the *PIN* family (involved in auxin efflux) were initially upregulated at around 25 d and then declined. The crucial factors in the auxin response pathway are the ARF and Aux/IAA proteins, and a previous study showed that *Arabidopsis* auxin response factor 1 (*AtARF1*) and *AtARF2* were weakly transcriptionally repressed or induced during senescence [34,39]. In our data, the expression of the *ARF1* and *ARF6* homologs decreased and that of the *ARF2* and *ARF4* homologs showed increased early and then declined during senescence. In addition, some other auxin response and signal DEGs were downregulated during senescence; e.g., homologs of small auxin upregulated RNA 12 (*SAUR12*), glycoside hydrolase family 3 (*GH3*) and auxin signaling f-box 2 (*AFB2*). These observations indicated a trend of decreasing auxin response at the gene transcription level as a result of changes in signaling proteins.

CKs have long been known as senescence-delaying hormones and are thought to be one of the key signals for the initiation of senescence [42-44]. Among genes related to CK signaling and responses, very few were differentially expressed in our dataset (Figure 6C). Among the CK-degrading oxidase/dehydrogenases [45], the cytokinin oxidase 5 (*CKX5*) homolog showed a slight decrease. CK levels could also be reduced during senescence by translocation into the vascular system. The phosphorus uptake 1 (purine permease 1, *PUP1*) homologs, members of the purine TP family, are involved in mediating CK uptake [46]. Most of the *PUP1* homologs were upregulated. For the CK signal transduction pathway, the homolog of *Arabidopsis* histidine phosphotransfer protein 2 (*AHP2*) was downregulated. *Arabidopsis* response regulators (*ARRs*), which are nuclear response regulators, act in the CK signaling pathway [47]. Of five B-type *ARRs* in our dataset, four *ARR12* homologs were downregulated during later stages of senescence. Interestingly, *ARR4* and *ARR8*, which belong to the A-type *ARRs,* were upregulated. These data suggested that CK might act as a negative regulator during cotton leaf senescence.

BRs play an essential role in several aspects of plant development and previous studies showed that BRs appear to promote developmental senescence [48,49]. Among BRs-biosynthesis genes, two cytochrome P450 72C1 (*CYP72C1*) homologs and one *CYP90A1* homolog were slightly upregulated (Figure 6D). This was consistent with earlier studies in *Arabidopsis* leaf senescence. For the BR signals, receptors and responses, the majority of altered transcripts were upregulated, including three BRI1-Associated Receptor Kinase 1 (*BAK1*) homologs, which were involved in BRs signaling, fifteen brassinosteroid insensitive 1 (*BRI1*) homologs, acts as BRs receptors, and two touch 4 (*TCH4*) homologs that are associated with BRs responses. *BKI1* acts negatively in the BR signaling by inhibiting *BRI1*’s activity [50,51], which was downregulated in our study.

ET has long been known as a major hormone that promotes leaf senescence [52]. In our study, a large proportion of ET-related key transcripts were upregulated (Figure 6E). In the ET-biosynthesis process, the homologs of 1-aminocyclopropane-1-carboxylic acid oxidase 4 (*ACO4*) were significantly upregulated at all leaf senescence stages, the 1-aminocyclopropane-1-carboxylic acid synthase 6 (*ACS6*) was significantly upregulated, while the *ACS10* gene was slightly upregulated at the early senescence stage. Among ET signal transduction-related genes, the ET receptor gene ethylene response 1 (*ETR1*) and ethylene insensitive 2 (*EIN2*) were not regulated, but several other components of ET signal transduction were significantly upregulated. The ET receptor gene *EIN4* that acts upstream of constitutive triple response 1 (*CTR1*) was continuously upregulated. *CTR1*, interacts with the ET receptors *ETR1* and ethylene response sensor (*ERS*), was also significantly upregulated. The TP-like RAN1 protein, which might be required to form functional ET receptors [53], and the downstream component *EIN3* were also up-regulated during leaf senescence. Meanwhile, several ET signal-related genes were upregulated, including the homologs of EIN3-binding F-box protein 1 (*EBF1*), ethylene response factor 2 (*ERF2*), *ERF3*, *ERF4*, *ERF5*, *ERF9*, *ERF12*, *ERF104*, and mitogen-activated protein kinase 4 (*MPK4*).

GAs comprises a group of growth hormones that control diverse processes, such as cell elongation, reproductive growth and senescence [54]. Previous reports showed that GAs are inhibitors of leaf senescence [55,56]. In our study, a large proportion of GA-related transcripts were downregulated (Figure 6 F), including two gibberellic acid-stimulated in *Arabidopsis* (*GASA1)*, two *GASA14* homologs, and seven alpha-expansin 1 (*EXPA1*) homologs that involved in GA-signaling, eight aquaporin tip growth defective1 (*TIP1*) homologs that belong to GA-induced genes and two GA receptors GA-insensitive dwarf 1 (*GID1*) homologs. In addition, many homologs of GA 2-oxidases and GA 3-oxidases were significantly upregulated during senescence.

SA has been well studied in stress responses, including disease and systemic acquired resistance. It was also reported that the SA has a role in leaf senescence [22,57]. A total of 154 genes associated with various aspects of SA-related pathways were identified (Figure 6G). The majority of transcripts were upregulated, including homologs of syntaxin of plants 121 (*SYP121*), necrotic spotted lesions 1 (*NSL1*), enhanced disease susceptibility 1 (*EDS1)*, nonexpressor of PR genes 1 (*NPR1*), phytoalexin-deficient 4 (*PAD4*) and NDR1/HIN1-like 25 (*NHL25*). Differential upregulation was most prominent for *PAD4* and *NHL25*. It was reported that *EDS1* and *PAD4* interact with each other, and that both genes are transducers of redox signals and important activators of SA signaling [58]. These data indicated that GA and SA might act as modulators of gene expression in senescing leaves.

JA regulates a range of plant growth and development processes, including seed germination, leaf senescence and plant defense against biotic and abiotic stresses [59,60]. Fourteen of 21 JA-biosynthesis genes were upregulated, including the homologs of defective in anther dehiscence1 (*DAD1*), lipoxygenase 3 (*LOX3*), allene oxide synthase (*AOS*), allene oxide cyclase 4 (*AOC4*), fatty acid desaturase 3 (*FAD3*) and OPC-8:0 CoA Ligase1 (*OPCL1*) (Figure 6H). Several genes that are supposedly involved in JA signaling or response were up- or down-regulated during senescence. For example, the homologs of jasmonate resistant 1 (*JAR1*), Jasmonate-ZIM domain proteins 6 (*JAZ6*) and coronatine insensitive 1 (*COI1*) were upregulated, while the homolog of *JAZ3* was downregulated. Collectively, these data indicated a coordinated upregulation of JA biosynthesis genes during leaf senescence in cotton.

## Discussion

### The utilization of diploid cotton genome is a good supplement for leaf senescence work in cotton

The whole genome sequence of *G. hirsutum* is currently not completed. The emergence and development of high-throughput sequencing technologies has helped the study of species with complex genomes (such as tetraploids), and have provided an unprecedented opportunity to examine the expression patterns of transcripts in specific tissues of interest. In this study, high-throughput Illumina sequencing technology combined with an advanced mapping strategy was used for expression profiling analysis of different developmental stages in cotton leaves. When mapping the DGE tags, we carefully considered the reference sequences, because the mapping percentages of tags were critical for downstream analysis *G. hirsutum* is a primary cultivated allotetraploid species and has a tetraploid genome (AD; 2n = 4 × = 52)], whereas, *G. raimondii* and *G. arboreum* are diploid cotton species and have D genome and A genome, respectively (D/A; 2n = 2× = 26) [61,62]. Initially, we just used the *G. raimondii* genome sequence for mapping tag sequences, about 23.6% of all distinct tags could be unambiguously mapped to a gene in the *G. raimondii* genome sequence, and about 45.2% of all distinct tags mapped to the genome (Additional file 7). It means only small part of tags could be assigned to genes. However, subsequently, we used two databases described in the methods to map the tag sequences, which obviously increased the mapping percentages of tag sequences. Thus, using two databases, but not only the *G. raimondii* genome sequence, for mapping tag sequences benefitted the downstream analysis.

### The initiation phase of leaf senescence in cotton

To obtain an insight into the timing and potential co-regulation of changes in genes and pathways in the complex process of leaf senescence in cotton, it was essential to observe sufficient leaf development stages. In this article, nine time points over 2 months of leaf development from young to full senescence were used. It has been reported that the well-established senescence markers include chlorophyll content, membrane ion leakage and gene expression, and that senescence symptoms usually start from the tip [1,33]. In the experiments reported here, the leaves started to show yellowing at the tip at around 35 d (Figure 1A); the chlorophyll levels reached a maximum at 25 d and then declined (Figure 1B). Chloroplast degeneration was accompanied by chlorophyll degradation and the progressive loss of proteins in the chloroplast, such as ribulose bisphosphate carboxylase (Rubisco) and chlorophyll a/b binding protein (CAB). Thus, leaf senescence is accompanied by decreased expression of genes related to photosynthesis (e.g., CAB) and protein synthesis (e.g., ribosomal protein small subunit (RPS), Rubisco (RBC), and by increased expression of SAGs. Some RBC and CAB genes were downregulated, and WRKY and NAC TFs were upregulated early, at around 25 d (Additional file 8). Considered together, according to the physiological characteristics and the expression pattern of these genes, we hypothesized that the initiation phase of leaf senescence in cotton under our growth condition was between 25 and 35 d.

### Comparative analysis of the SAGs with other plants

Previous studies have identified many SAGs during plant senescence. The most recent version of the leaf senescence database (LSD, June, 2013; http://www.eplantsenescence.org/) documented 5,356 SAGs from 44 plant species; the majority (over 3,745) of these were identified in *Arabidopsis*. These SAGs encode a wide variety of proteins with diverse functions, including macromolecule degradation, nutrient recycling, transcriptional regulation, and hormone response pathways. In our experiment, 3,624 DEGs were identified during developmental senescence in cotton. However, the extent of the similarity or difference of the molecular mechanisms underlying senescence between cotton and other species is unknown. To address this question and analyze SAG orthologs between cotton and other plants, all identified DEGs were compared with those in other species in the leaf senescence database. Based on this database, 2,414 (66.6%) out of 3,624 DEGs have homologous genes in other plants in the LSD, whereas, 1, 210 DEGs generated in this study were not highly homologous to known SAGs in the LSD. This implied that these genes might be specific to cotton during leaf senescence. These novel SAGs were searched against a variety of databases, and approximately 50% of the genes remained of unknown function, which could be considered as putative cotton-specific sequences [63-67]. However, many matched known function genes are involved in photosynthesis (e.g., photosystem I/ II reaction center subunit, light-harvesting complex II protein), chloroplast degeneration (e.g., chlorophyll a/b binding protein), ribosomal protein, metabolic process protein, and zinc finger protein. Therefore, these novel SAGs are candidate target genes for future modelling of senescence transcriptome in cotton.

### The TFs are highly enriched during leaf senescence

Several previous studies have highlighted the crucial role of TFs in plant leaf senescence. In the present study, 519 TFs were significantly enriched among the DEGs identified during cotton leaf senescence (Figure 5 and Additional file 5). Among the various TFs, WRKY, bHLH, C3H, NAC, and AP2-EREBP were the top five significantly upregulated TFs during leaf senescence of cotton. WRKY TFs have been reported to be important for senescence [68,69], and are downstream of defense signaling mitogen-activated protein kinase pathways, which are involved in the regulation of JA and SA dependent defense signaling pathways [70,71]. Several AP2-EREBP families modulate responses to leaf senescence-associated signaling molecules, such as ROS, ethylene, JA, ABA, and CK [72,73]. For some of these, there is a striking overlap with the senescence-associated TFs in *Arabidopsis*: the WRKY, NAC, C3H and AP2-EREBP families. The apparent conservation of the regulatory mechanisms behind the process of senescence across distantly related species is rather encouraging. It suggested that these TFs may lead to fundamental clues about the regulation of senescence. However, there were considerable differences. The expression patterns of these TFs gene were different. For example, some MYB TFs have been shown to participate in leaf senescence, drought stress and disease resistance [74-76], which were significantly upregulated during leaf senescence in our study, but there were no significant changes in *Arabidopsis.* These data suggested that different regulatory patterns existed in cotton leaf senescence.

### Regulation of plant hormone related pathways during senescence

For many years, plant hormones have been thought to play important regulatory roles in either promoting or delaying leaf senescence [77]. For example, ET, ABA and JA are regarded as promoters of senescence, whereas CKs have been observed to delay the progress of senescence. In this study, many genes related to hormone pathways showed differential expression during senescence (Figure 6). The results gave a clear picture with regard to the up or downregulation of genes associated with various hormones. As one of the main plant hormones, ABA has been implicated in the regulation of environmental stresses and senescence [35]. In *Arabidopsis*, the genes encoding the key enzymes in ABA biosynthesis were upregulated during senescence, such as *AtNCED*, *AtAAO1* and *AtAAO3* [12,78]. In our data, key ABA-biosynthesis genes showed no significant changes; only the homolog of *AtNCED5* was slightly upregulated at the early senescence stage, around 35 d (Additional file 6). However, many ABA glucosyltransferases, which are involved in ABA catabolism, were significantly upregulated, such as *UGT73B3*, *UGT74D1*, *UGT75B1* and *UGT75B2*; while the ABA-inactivating glucosylase*UGT71B6* were downregulated (Figure 6A). In addition, the key ABA signal transduction related genes, including the core group of ABA receptor PYR/PYL family proteins and the type 2C protein phosphatase, were significantly upregulated, which indicated that the ABA signaling pathway is active during leaf senescence. The ABA inducible receptor-like kinase gene of *Arabidopsis*, RPK1, as a positive regulator of senescence in *Arabidopsis* Leaves [79], was also significantly upregulated during leaf senescence. Therefore, we predicted that ABA may be regarded as a hormonal trigger of leaf senescence, by maintaining the certain ABA level and upregulating ABA signaling.

Although a delaying effect on senescence by auxin was reported many years ago [35], the function of this hormone in senescence is poorly understood compared with other hormones, such as ABA, ET, JA and CK. Previous studies showed that indole acetic acid (IAA) biosynthetic genes encoding tryptophan synthase (*TSA1*), IAAld oxidase (*AO1*), and nitrilases (*NIT1-3*) are upregulated during age-dependent leaf senescence [12], whereas these genes showed no significant changes in our data (Additional file 6). Many auxin efflux carrier family genes were strongly upregulated, and some genes that play essential roles in auxin signal transduction were significantly differentially expressed (Figure 6B). This suggested that changes in auxin signaling and transport, rather than the endogenous auxin level itself, could be important in modulating senescence. Information on CKs’ involvement in the regulation of senescence in cotton is sparse. A few genes involved in CK transport was upregulated, however, several CK signaling genes, such as the homologs of *AHP2* and *ARR12*, appeared to be downregulated. Thus we presumed that leaf senescence is not so closely related to CK regulation under our growth conditions as other hormones.

Few previous studies have demonstrated the role of GA in leaf senescence. Some bioactive GAs, such as GA1, GA4, and GA53, can be deactivated by GA 2-oxidases, e.g. Gibberellin 2-oxidase 7 (*GA2ox7*) and *GA2ox8* in plants [80-83]. Thus, GA 2-oxidation is a well-characterized type of GA catabolism that is involved in the deactivation of GA. In our data, many homologs of GA 2-oxidases and GA 3-oxidases were significantly upregulated, such as *GA2ox1*, *GA2ox2*, *GA2ox8*, *GA3ox1* and *GA3ox3* (Figure 6F and Additional file 6). This suggested that during senescence at least some biologically active gibberellins are deactivated. These results are consistent with those obtained from *Arabidopsis* [12]. The GA signal is received and transduced by the GID1 GA receptor/DELLA repressor pathway [84]. RGL1 and RGL2 belong to the DELLA group of proteins, which are negative regulators of the GA signaling pathway [85,86]. Two homolog of *RGL2* were significantly upregulated. This indicated that DELLAs repressed the GA signal. In addition, several other GA signaling proteins were significantly downregulated, such as the homolog of *GASA1*. Some GA-responsive genes also displayed senescence-associated regulation. A well-known example is the GA-induced aquaporin, *TIP1.1* [87], which was downregulated. However, the homologs of GA-responsive xyloglucan endotransglycosylase *MERI5* were upregulated during leaf senescence. Studies on *TIP1.1* and *MERI5* revealed that there is cross talk between the GA and BR pathways. GA and BR regulate *AtTIP1.1* antagonistically, whereas *MERI5* was is positively and additively regulated by either GA or BR treatment [88]. During leaf senescence, GA activity appears to be reduced; therefore, a different regulator, possibly BR, effects the senescence-associated induction of *MERI5*.

BRs play an essential role in diverse developmental programs, including leaf senescence, and several evidences have revealed their role in promoting leaf senescence [49]. Some BR biosynthesis-related genes were slightly upregulated, and some others were downregulated (Figure 6D and Additional file 6), suggesting that BR biosynthesis does not significantly increase during leaf senescence. In addition, *BIN2*, *BES1* and *BZR1* showed no significant change in expression, which act as the central transmitters of the BR signal from the receptors to the target genes. *BRL1* and *BRL3* act as BRs receptors, supposedly functioning specifically in provascular differentiation [89]. However, most of the *BRL1* and *BRL3* homologs were upregulated during leaf senescence, which suggested that BRs might modulate the properties of the vascular tissue in senescing leaves toward efficient export of metabolites.

ET acts as a major hormone in hastening senescence [52]. The ET biosynthetic genes encoding ACC synthase (*ACS*), ACC oxidase (*ACO*), and nitrilase are upregulated in senescing leaves [12]. During the process of ethylene metabolism, Arabidopsis ethylene overproducer 1 (*ETO1*) affected the posttranscriptional regulation of *ACS* by inhibiting ACS enzyme activity and targeting it for protein degradation. This permits rapid modulation of the concentration of ET [90]. Furthermore, *ACS* is substrates of *MPK3*/*MPK6*, and phosphorylation of *ACS2* and *ACS6* by *MPK3*/*MPK6* leads to the accumulation of ACS protein, and thus, elevated levels of cellular ACS activity and ethylene production [91,92] In the present study, the homolog of *ACS10*, which catalyze the rate-limiting step in the ET biosynthesis pathway, was slightly upregulated at the early stage of leaf senescence (Figure 6E and Additional file 6). Coupled with the significant upregulation of the homologs of the *MPK3*/*MPK6* genes, the data suggested that the ACS activity was maintained at a high level during leaf senescence in cotton. In the subsequent step, *ACC* is converted to ET by *ACO*, which was also upregulated. Thus, the expression profiles of this set of proteins imply an increase in ET production in senescing leaves. ET signal transduction supposedly follows a “linear” pathway, with membrane-bound receptors at the beginning, multiple positive and negative regulators in between, and TFs at the end of the chain. The expression of ET receptor *ETR1*, *ETR2* and *ERS1* were not significantly regulated. *EIN3*/*EIN4*, as the downstream components of the ET receptor showed increased expression. Meanwhile, one immediate target of *EIN3* is *ERF1* [93], which appeared to be upregulated. Several other ERF proteins were also upregulated but with different expression profiles. Moreover, three EIN3-binding F box proteins were also upregulated. Taken together, the data suggested that ET may act as a positive regulator in leaf senescence in cotton.

SA is involved in pathogen response and pathogen-mediated cell death. It was also reported that the SA-signaling pathway is active in the control of gene expression during developmental senescence [57]. In our study, many SA signaling genes were significantly upregulated. For example, two homologs of *NHL25*, one homolog of *NPR1* and two homologs of *PAD4* were significantly upregulated (Figure 6G). Scarecrow-like 14 (*SCL14*), a member of the *GRAS* family of regulatory proteins, interacts with the TGA2 transcription factor and affects the transcription of stress-responsive genes [94]. We observed that all four homologs of this gene were upregulated. In addition, the expression profiles of many SA-responsive genes were also upregulated, such as the homolog of the *AF2* gene, accelerated cell death 6 (*ACD6*). Moreover, as mentioned before, many WRKY TFs involved in the regulation of SA-dependent defense signaling pathways were upregulated, which revealed that SA has important roles in positively regulating leaf senescence in cotton by interacting with the WRKY family.

JA functions in the induction of leaf senescence in many plant species [60,95] by regulating the expression of various SAGs [22]. Here, JA biosynthesis genes showed a significantly increase in expression almost the entire process of senescence, including *LOX3*, *AOS*, *AOC4* and *OPR3*. The COI1, an F-box protein, as a key component of the receptor complex in the JA signaling pathway [96,97], were also up-regulated during senescence. This protein forms part of an enzyme complex called SCF^COI1^. JAZ proteins are ubiquitinated via SCF^COI1^ in response to JA, they function as repressors of the JA signaling pathway, and recent investigations revealed that COI1 (in the SCF^COI1^ complex) and several JAZ proteins form a coreceptor complex [98,99]. The genes implicated in controlling JA responses are upregulated, including *JAZ6*, *JAZ7* and *MYC2*. JAZ proteins have been shown to bind to MYC2 directly and prevent its action [100]. Thus, it indicated that the JA signaling was active at the very early stage during leaf senescence, and these data also provide additional evidence for JA as a modulator of gene expression in developmental leaf senescence.

### Transcriptome dynamics during leaf senescence

As expected, we identified groups of genes that are co-expressed and hence may be co-regulated across the entire time series; however, it is very important to identify an ordering of events across the leaf developmental stages. It is clear that there are progressive changes in genes being upregulated or downregulated as senescence progresses, and these are highly informative in indicating changes in metabolic pathways. Genes upregulated from the first time point (15 d), before the leaf is fully expanded, were enriched for response to biotic stimulus, response to endogenous stimulus, response to abiotic stimulus, response to stress. In addition, genes involved in both JA and SA responses were enhanced in expression before 25 d, indicating that there may be a role for these hormones even before the leaf is fully expanded. JA-related genes upregulated at this time point include genes required for JA biosynthesis such as two *LOX*, two *AOC*, *OPR*, and *OPCL* (Additional file 6). Genes implicated in JA responses and signalling were upregulated, including *JAZ6*, *JAZ7*, and *JAR1*. SA biosynthetic genes showed no significantly increase in senescence, only some SA metabolism genes such as UDP-glycosyltransferase 74F, *UGT74F1* and *UGT74F2* were regulated. The SA glycosides are actively transported from the cytosol to the vacuole as an inactive storage precursor that can later be converted back to SA [101]. Besides, several JA-signaling genes (e.g., *NHL25* and *PAD4*) were upregulated before 25 d. This suggested that these two hormones modulated leaf senescence primarily by responding to environmental cues and stress at the early stage of senescence. Tetrapyrroles are essential components of critical biological processes, including respiration and photosynthesis; many tetrapyrrole or chlorophyll biosynthesis genes are first downregulated at 25 d. Thus, the requirement for de novo chlorophyll biosynthesis appears to cease in stage of leaf maturity, indicating that all chloroplasts are fully developed. Genes involved in ribosome and ribosomal protein genes were downregulated at 25 d, indicating that expansion of the ribosomal content of the cells has slowed down. Also, downregulated genes were significantly enriched for genes linked to the plastid, vacuole and thylakoid, and with functions in metabolic processes, particularly photosynthesis, lipid biosynthetic process and fatty acid biosynthetic process at this stage. It indicated that photosynthetic activity probably started to drop at this stage. Down-regulation of these groups of genes reflected the shut down of cellular biosynthetic activity at the early senescence stage. Moreover, at 25 d, within the molecular function annotations, transferase activity, catalytic activity, kinase activity, oxygen binding, calmodulin binding and transcription regulator activity were overrepresented, whereas there was significant enrichment for protein metabolic process and protein modification process in the biological process terms, reflecting kinase-signalling cascades are likely to function and the considerable degradation that is underway.

At the next time point (35 d), there is an overrepresentation of genes involved in macromolecule metabolic process, response to ABA stimulus, regulation of defense response. Similarly, genes involved in ribonucleotide binding, ATP binding, protein binding and lipase activity are up regulated. The previous investigations showed that ABA is a key hormone mediating plant responses to environmental stresses, and ABA induces the expression of antioxidant genes and enhances the activities of antioxidative enzymes that play a role in protecting the cellular functions [102]. ABA-related genes upregulated at this time point include genes required for ABA signal transduction such as *PP2C* and *RPK1*. It appeared that ABA may control activities of both the cellular protection activities and senescence activities at the early stages of senescence. By the later stage of senescence, many genes encoding TFs are obviously upregulated, including WRKY, NAC and C3H family. Genes for response to chemical stimulus also upregulated, some genes annotated ET related pathway, i.e., *ACS6*, *ACO4*, *ERF12* and *ERF104* being upregulated late, indicating that the ET regulation of senescence may have a significant role at this time. Once the senescence process is initiated, massive degradation and recycling of macromolecule occurs during this stage. The leaf was converted from a source of photosynthetic carbon to mobilizable nutrients, as cellular components become degraded and mobilized from the leaf. These results are of great importance for future modeling of senescence transcriptional networks. However, the exact role of these regulators and the cross-talk between them to fine-tune the leaf senescence in cotton remains undiscovered.

## Conclusions

To the best of our knowledge, this study is the first to reveal genome-wide multiple time-course transcriptome dynamics during leaf senescence in cotton. In total, 3,624 DEGs were identified and analyzed for their potential role in cotton leaf development using clustering, GO enrichment and KOBAS analysis. Furthermore, 519 differentially expressed TF transcripts from 50 TF families were identified; some of these TFs have been reported to be critical components of plant adaptive response to biotic, abiotic stresses and senescence. Eight hormone-related differentially expressed genes were identified, of which many genes involved in the ET, BR, JA, SA and ABA pathways were upregulated, indicating that these hormone-related genes might act as positive regulators in cotton leaf development and senescence. However, most auxin and GA pathway-related genes were downregulated, suggesting that these two hormones may act as negative regulators of senescence. Thus, our study revealed a large set of candidate genes that may play important roles in leaf development and senescence in cotton. Meanwhile, the detailed investigations of the pathways and candidate genes identified in this study would not only be useful for enhancing our understanding of the molecular mechanism involved in leaf development and senescence of *G. hirsutum*, but also may provide potential targets for crop improvement via breeding and reverse genetics approaches.

## Methods

### Plant material preparation and RNA extraction

*G. hirsutum* cv. CCRI 36 was grown in a cotton climatic chamber at 30/25 ± 3°C under a light/dark cycle of 12/12 h. Similar sized emerging fourth true leaves were tagged. The day when emerging true leaf 4 was expanded was considered the first day. Sampling of leaves started on the 5^th^ day and continued every 10 days until full senescence was reached. At each time point, three biological replicates were designed; the samples of each biological replicate were pooled from 10 plants, the plants being randomly selected to avoid any potential effects of position within the growth room. The three biological replicates were mixed divided into two parts, one was used for RNA isolation for Illumina sequencing (only one mixed sample was used in each developmental stage in sequencing.), and the other was used for chlorophyll and malondialdehyde (MDA) measurements. An RNA prep Pure Plant Kit (TIANGEN BIOTECH) was used to isolate the total RNA, according to the manufacturer's protocol.

### MDA and chlorophyll measurements

Chlorophyll was extracted using 80% (v/v) acetone, vortexed, and then stored at 4°C overnight in the dark. The samples were then centrifuged for 2 min at 12,500 × g, and the absorbance of 1 mL of supernatant was measured at 663 and 645 nm using the method described by Lichtenthaler [103]. MDA contents were measured by the 5% thiobarbituric acid reaction, as described by the procedure of Saher [104].

### DGE sequencing and analysis

Approximately, 8 μg of total RNA was used. Tag libraries were prepared using the Illumina Gene Expression Sample Prep Kit, following the manufacturer’s protocol, as described by Luan et al. [105]. The libraries were then sequenced using an Illumina HiSeq™ 2000 with 50-bp single-end (SE) reads each. To increase the mapping percentages of tag sequences, two different reference databases were employed, which included *G. raimondii* genome and *G. arboretum* genome. In order to remove redundancy, these two genome sequences were merged into one reference database according to following procedure: all CATG + 17-nucleotide tag sequences from the 3’-most CATG to the poly(A)-tail of the predicted coding sequences from the genome data of *G. raimondii* and *G. arboretum* were created, all these tags were allowed no more than one nucleotide mismatch to remove redundancy; if two or more tags from different genome sequences are the same, *G. arboretum* genome sequence were selected with higher priority. The remaining genome sequences were used as reference sequence for DGE tag mapping. Then, the preprocessed database of all possible CATG + 17-nucleotide tag sequences was generated using reference sequence. All tags were allowed no more than one nucleotide mismatch. Clean tags mapping to reference sequences from multiple genes were filtered out, and the remaining clean tags were designated as unambiguous clean tags. For gene expression analysis, the number of unambiguous clean tags for each gene was calculated and then normalized to TPM (number of transcripts per million clean tags) [106,107].

### Validation of gene expression profile using qRT-PCR

To validate the results of the DGE-based analyses, the expressions of 25 genes that were randomly selected based on different expression levels in DGE-based data were measured by qRT-PCR. Importantly, the samples used for RNA isolation in qRT-PCR experiments were different from the samples used in RNA-Seq analysis. The RNA samples were prepared using the same method mentioned above. cDNA was reverse transcribed from RNA by PrimeScript® RT reagent Kit with gDNA Eraser (Takara, Biotechnology Co., Ltd.) using an Oligo dT Primer and random hexamer as RT primers, according to the manufacturer’s protocol. The specific primers for the selected genes and internal control gene (actin) are listed in Additional file 9. Samples were run in technical triplicate on each plate using the SYBR Green PCR Master Mix (Takara) on an ABI 7500 Real-time PCR System (Applied Biosystems, USA) following the manufacturer’s instructions. The relative expression levels were calculated by the comparative 2^−△△Ct^ method [108]. The results were normalized to the expression level of actin and relative to the 15 d sample. The Pearson correlation coefficients of the expression patterns of selected genes between qRT-PCR and DGE were calculated using the SAS software.

### Identification and analysis of DEGs

To identify DEGs across the six different time points, pair wise comparisons among the six samples were performed using a rigorous algorithm method based on a previous method [109]. The DEGs were obtained after filtering using a threshold false discovery rate (FDR) of ≤0.001 and an absolute value of log2Ratio ≥2. Two-fold change has been generally accepted as a significant or meaningful change in DGE data analyses; however, we used four-fold change as the cut-off for more rigorous analysis in this study. Additionally, one of the two samples was required to have a raw intensity of gene (raw mapped tags) ≥10 in the pairwise comparison. MultiExperiment Viewer (MeV, v4.7.4) [110] produced the cluster analysis of the DEGs, based on the K-means method [111]. MeV performed the clustering analysis of co-regulated genes expression patterns based on hierarchical clustering. For GO annotation enrichment, the best hit corresponding to each cotton transcript was identified using a BLAST search. The BiNGO tool preformed the GO enrichment analysis of gene clusters, as described by Maere [30]. KOBAS was then used to identify biochemical pathways involved in cotton leaf senescence and to calculate the statistical significance of each pathway with the default criteria [112]. KOBAS uses Fisher’s exact test to identify significant pathways and then performs an FDR correction to reduce type-1 errors. Pathways with corrected P values <0.05 were considered statistically significant.

To identify genes encoding for TFs among the DEGs, all DEGs were compared with protein sequences download from a comprehensive plant TF database, PlnTFDB [113] using Blastx with an E-value ≤ 10^−10^. The HMMER (E-value ≤ 10^−10^, http://hmmer.janelia.org/) program was used to search against the BLAST search results using the Pfam program (http://pfam.sanger.ac.uk/). Finally, we manually checked the search results to reduce false positives. For analysis and classification of hormone-related genes, all DEGs were compared with the protein sequences from the Arabidopsis Hormone Database (http://ahd.cbi.pku.edu.cn/) using Blastx (E-value ≤ 10^−10^).

### Availability of supporting data

Raw Illumina reads have been deposited into NCBI’s SRA (sequence read archive), the accession number is SRR656612.

## Additional files

Additional file 1:
**List of gene differentially expressed during cotton development and senescence.**
Additional file 2:
**List of leaf senescence orthologs between cotton and other species.**
Additional file 3:
**Significantly enriched GO terms in each cluster.**
Additional file 4:
**All enriched pathways in cotton identified by KOBAS during leaf development and senescence.**
Additional file 5:
**List of transcription factors differentially expressed during leaf senescence.**
Additional file 6:
**List of eight plant hormone pathway-related genes.**
Additional file 7:
**Tag analysis statistics by using** ***G. raimondii*** **genome sequence.**
Additional file 8:
**List of some senescence-associated genes (SAGs) whose differential expression started at 25 d during leaf senescence.**
Additional file 9:
**List of primers used for qRT-PCR.**

## Abbreviations

SAGs: Senescence-associated genes
TFs: Transcription factors
Bt: Bacillus thuringiensis
CK: Cytokinin
ABA: Abscisic acid
MDA: Malondialdehyde
DGE: Digital gene expression
DEGs: Differentially expressed genes
LSD: Leaf senescence database
MeV: MultiExperiment Viewer
KEGG: Kyoto Encyclopedia of Genes and Genomes
KOBAS: KEGG Orthology Based Annotation System
ET: Ethylene
BR: Brassinosteroid
JA: Jasmonic acid
SA: Salicylic acid
UGT: UDP-glucosyltransferase
ABA2: ABA deficient 2
PYR: Pyrabactin resistance
PYL: PYR1-like
PP2C: Protein phosphatase 2C
ADAP: ARIA-interacting double AP2 domain protein
PUB44: Plant U-box 44
CYP75B1: Cytochrome P450 75B1
PGP1: P-glycoprotein 1
PIN8: Putative auxin efflux carrier component 8
ARF1: Auxin response factor 1
SAUR12: Small auxin upregulated RNA 12
GH3: Glycoside hydrolase family 3
AFB2: Auxin signaling f-box 2
CKX5: Cytokinin oxidase 5
PUP1: Purine permease 1
AHP2: Arabidopsis histidine phosphotransfer protein 2
ARRs: Arabidopsis response regulators
BRI1: Brassinosteroid insensitive 1
BAK1: BRI1-Associated Receptor Kinase 1
TCH4: Two touch 4
ACO4: 1-aminocyclopropane-1-carboxylic acid oxidase 4
ACS6: 1-aminocyclopropane-1-carboxylic acid synthase 6
ETR1: Ethylene response 1
EIN2: Ethylene insensitive 2
CTR1: Constitutive triple response 1
ERS: Ethylene response sensor
EBF1: EIN3-binding F-box protein 1
ERF2: Ethylene response factor 2
MPK4: Mitogen-activated protein kinase 4
GASA1: Gibberellic acid-stimulated in Arabidopsis
EXPA1: Alpha-expansin 1
TIP1: Tip growth defective1
GID1: GA-insensitive dwarf 1
SYP121: Syntaxin of plants 121
NSL1: Necrotic spotted lesions 1
EDS1: Enhanced disease susceptibility 1
NPR1: Nonexpressor of PR genes 1
PAD4: Phytoalexin-deficient 4
NHL25: NDR1/HIN1-like 25
DAD1: Defective in anther dehiscence1
LOX3: Lipoxygenase 3
AOS: Allene oxide synthase
AOC4: Allene oxide cyclase 4
FAD3: Fatty acid desaturase 3
OPCL1: OPC-8:0 CoA Ligase1
JAR1: Jasmonate resistant 1
JAZ6: Jasmonate-ZIM domain proteins 6
COI1: Coronatine insensitive 1
Rubisco: Ribulose bisphosphate carboxylase
CAB: Chlorophyll a/b binding protein
RPS: Ribosomal protein small subunit
RBC: Rubisco
IAA: Indole acetic acid
TSA1: Tryptophan synthase
AO1: IAAld oxidase
NIT1-3: Nitrilases
GA2ox7: Gibberellin 2-oxidase 7
ETO1: Ethylene overproducer 1
EBF: EIN3-binding F box proteins
SCL14: Scarecrow-like 14
ACD6: Accelerated cell death 6
